# Elucidation of an intrinsic parameter for evaluating the electrical quality of graphene flakes

**DOI:** 10.1038/s41598-018-37010-x

**Published:** 2019-01-24

**Authors:** Ha-Jin Lee, Ji Sun Kim, Kwang Young Lee, Kyung Ho Park, Jong-Seong Bae, Mahfuza Mubarak, Haeseong Lee

**Affiliations:** 1 Western Seoul Center, Korea Basic Science Institute, 150 Bugahyun-ro, Seoudaemun-gu, Seoul 03759 Republic of Korea; 2 Department of Chemistry and Nano Science, Ewha Womans University, 52 Ewhayeodae-gil, Seoudaemun-gu, Seoul 03760 Republic of Korea; 3 Department of Carbon and Nanomaterials Engineering, Jeonju University, 303 Cheonjam-ro, Wansan-gu, Jeonju 55069 Republic of Korea; 4 Korea Advanced Nano Fab Center, 109 Gwanggyo-ro, Yeongtong-gu, Suwon 16229 Republic of Korea; 5 Busan Center, Korea Basic Science Institute, 30 Gwahaksandan 1-ro 60beon-gil, Gangseo-gu, Busan 46742 Republic of Korea

## Abstract

A test method for evaluating the quality of graphene flakes, such as reduced graphene oxide (rGO) and graphene nanopowder (GNP), was developed in this study. The pelletizer was selected for a sampling tool, which enables us to formulate the flake sample as a measurable sample. Various parameters were measured from the pelletized sample in order to elucidate the best parameter for representing the quality of the graphene flakes in terms of their electrical properties. Based on the analysis of 4-probe measurement data on the pelletized sample, the best intrinsic parameter is volume resistivity (or volume conductivity) rather than resistivity (or conductivity). Additionally, the possible modification of a sample before and after the pressurization was investigated by electron microscopy and Raman spectroscopy. No significant modification was observed. The volume conductivity in the two types of the graphene was different from their individual conductivities by one order of magnitude. Based on the results of X-ray photoelectron spectroscopy and Raman spectroscopy measurements, the volume conductivity of the graphene flake samples was governed by the oxygen content in the sample. Our achievements will promote the effective use of powder-type graphene products for further applications.

## Introduction

Graphene has attracted much attention due to its extremely high mobility and ballistic transport of electrons, which enable its utilization as a next-generation electronic material^1,2^. To date, two types of graphene, namely, films and powders have been developed for specific applications. The film-type graphene fabricated by the chemical vapour deposition (CVD) or epitaxy methods has been studied for replacing the indium-tin oxide (ITO) glass that is widely used in the current display industry^3–5^. Furthermore, graphene films can be used to fabricate flexible transparent conducting films that can be used as flexible substrates in printed electronics or wearable electronics^5–7^. The powder-type graphene that is mainly produced by reduction of graphite oxide monolayer is the other type of graphene that may enable more practical applications, for example serving as an energy material with a high density as well as an outstanding electrical conductivity^8–10^. The use of powder-type graphene can be extended to the formation of functional composites due to its lower density and high mechanical properties^11,12^.

During the first decade after the discovery of graphene, most work in academic and industrial research has been done on the film-type graphene. However, technical obstacles must still be overcome to enable the economically viable use of film-type graphene as a transparent conducting electrode in display industry because currently, homogeneous electrical conductivity cannot be obtained over the entire surface^13,14^. Recently, more effort has been made in utilizing the powder-type graphene in industrial applications due to another advantage of this novel material. Since powder-type graphene exhibits greater versatility for use with other matrices, it can be a good candidate for use as an anodic material for secondary batteries, as an additive to activated carbon for supercapacitors, and in other applications^15,16^. In particular, the introduction of chemical exfoliation for the fabrication of powder-type graphene can reduce its production cost and enhance large scale productivity, possibly accelerating the use of powder-type graphene product in industrial applications in the near future^8,17–19^.

Since powder-type graphene is expected to be widely used as mentioned above, there is high demand from both the manufacturers and users for developing evaluation methods that can be applied to the powder-type graphene products^20^. As the nature of powder-type graphene is arguable, it is essential to define the term and evaluate the material. Fortunately, the discussion on these issues have been actively made by several technical groups, especially by standardization societies such as ISO (International Organization for Standardization) and IEC (International Electrotechnical Commission). In 2017, ISO published the technical specification (TS) which defines graphene and classified the graphene in terms of geometric factors^21^. In this TS graphene is categorized by four types such as graphene, bilayer graphene, few-layer graphene, and graphene nanoplatelet. The controversy on the definition of graphene nanoplatelet is forced to use graphene flake^20^, which is widely used, instead of powder-type graphene here-in-after in this paper to our best knowledge.

Since the electrical properties of graphene flakes are simultaneously correlated to its geometric and electronic characteristics^22^, it is necessary to select the parameter that best represents the electrical quality of graphene flakes. Graphene flakes are generally produced by the exfoliation of graphite in an acidic solution^8,17,18^. In its manufacturing process, a layer of graphene is fabricated via oxidation. Hence, a reduction process should be used after the oxidation process to produce a powder product which is the so-called reduced graphene oxide (rGO). Consequently, the electrical properties of such graphene are influenced by its oxygen amount and flake size^23,24^. Therefore, it is necessary to identify suitable sample preparation and measurement conditions for obtaining the target properties.

Generally, the powder resistivity measurement method is used to investigate the electrical properties of the powder samples^25,26^. To measure the electrical properties, it is necessary for the powder sample to be formulated as a pellet under pressurization. As the applied pressure is varied, two parameters such as the resistance and thickness of the pellet are directly measured at the given pressure^25–28^. The sheet resistance is calculated by considering an instrument-dependent correction factor (*F*)^29,30^. From the sheet resistance data, the resistivity of the sample is calculated by taking the obtained thickness value into account. Since the resistivity or the conductivity of a sample is an intrinsic property, it should be equal or close to that of the CVD–grown graphene. Another candidate parameter to should be considered is volume resistivity or volume conductivity which is a function of the given pressure^26^. Accordingly, we selected the following parameters to determine how well they represent the quality of graphene flakes in this study: (i) sheet resistance (or conductance), (ii) resistivity (or conductivity), and (iii) volume resistivity (or volume conductivity). Based on a systematic review of the results for these three parameters obtained for different types of graphene samples, the best parameter for the characterization was determined.

In addition to electrical characterization of the flakes under pressure, the possible modifications of the sample structure before and after the pressurization were examined by electron microscopy and Raman spectroscopy. The quantitative and qualitative analysis of carbon and oxygen, which are the main components in the samples, was also performed using X-ray photoelectron spectroscopy (XPS). These results will be used for evaluating the electrical quality of the graphene products and for determining the key characterization parameters in order to extend their applications.

## Results and Discussion

**Figure 1:**
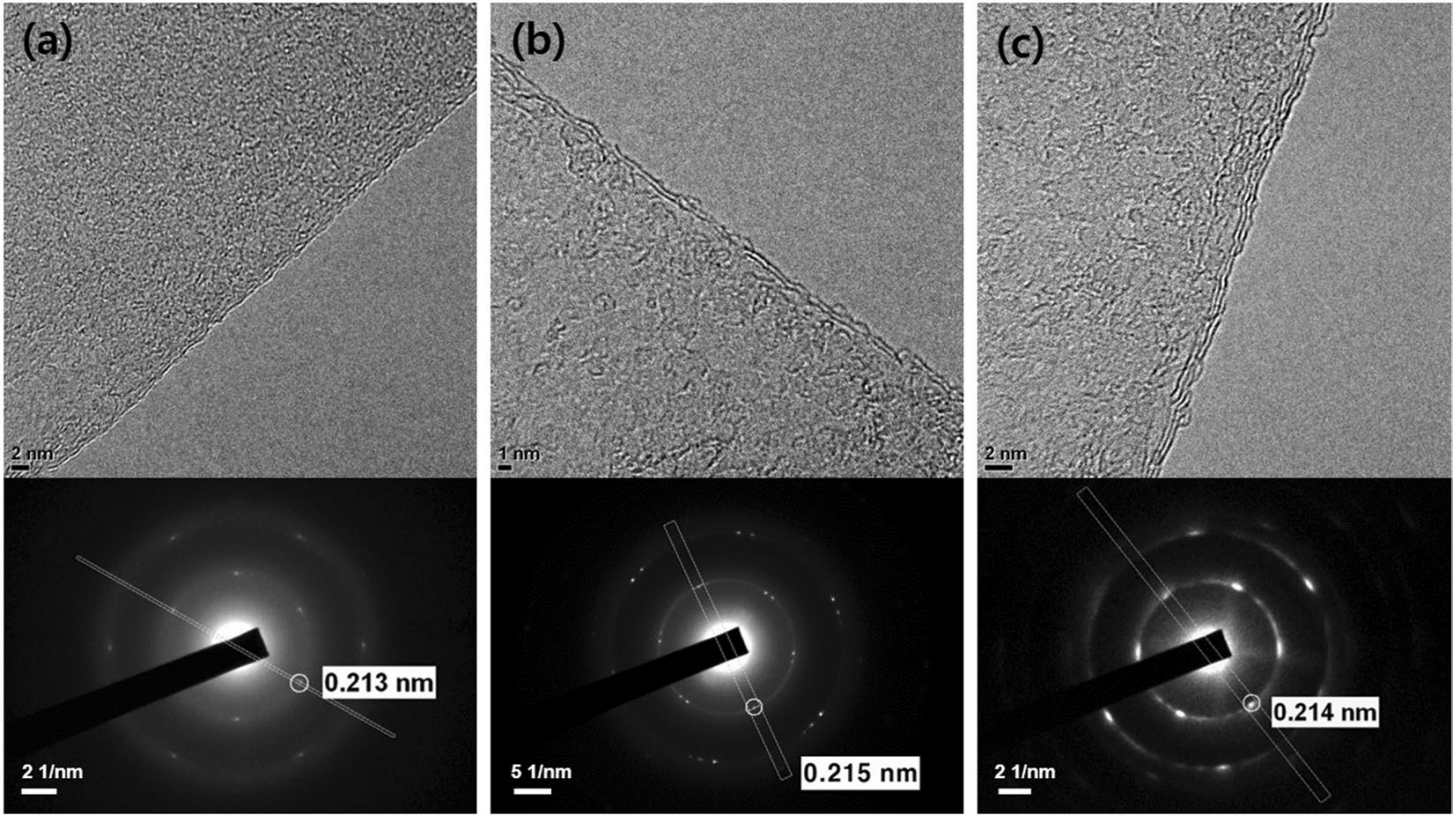
TEM images (top) and SAED patterns (bottom) of (**a**) rGO-A, (**b**) rGO-B, and (**c**) GNP.

Three types of graphene flakes were used in this study, namely, i) two commercialized brands labelled as rGO by their manufacturers and ii) graphene nanopowder (GNP) which is also commercially available. Since the number of layers, which is indicated by the thickness, is a key parameter of a graphene product, the thickness data provided in the material specifications of these products were confirmed by transmission electron microscopy (TEM). High-resolution TEM images and selected area electron diffraction (SAED) patterns of the as-received graphene materials are shown in Figs 1 and S1. Figure 1a,b show the structures of the two rGOs and reveal that they contain mainly one or two layers of graphene while the GNP image indicates the presence of graphene with at least three layers in this product (Fig. 1c). From the SAED data of the three products shown in Fig. 1 and 1S (bottom), the existence of the (0–110) plane of graphene was confirmed by the hexagonal diffraction pattern and the 0.213~0.215 nm spacing^31^.

**Figure 2:**
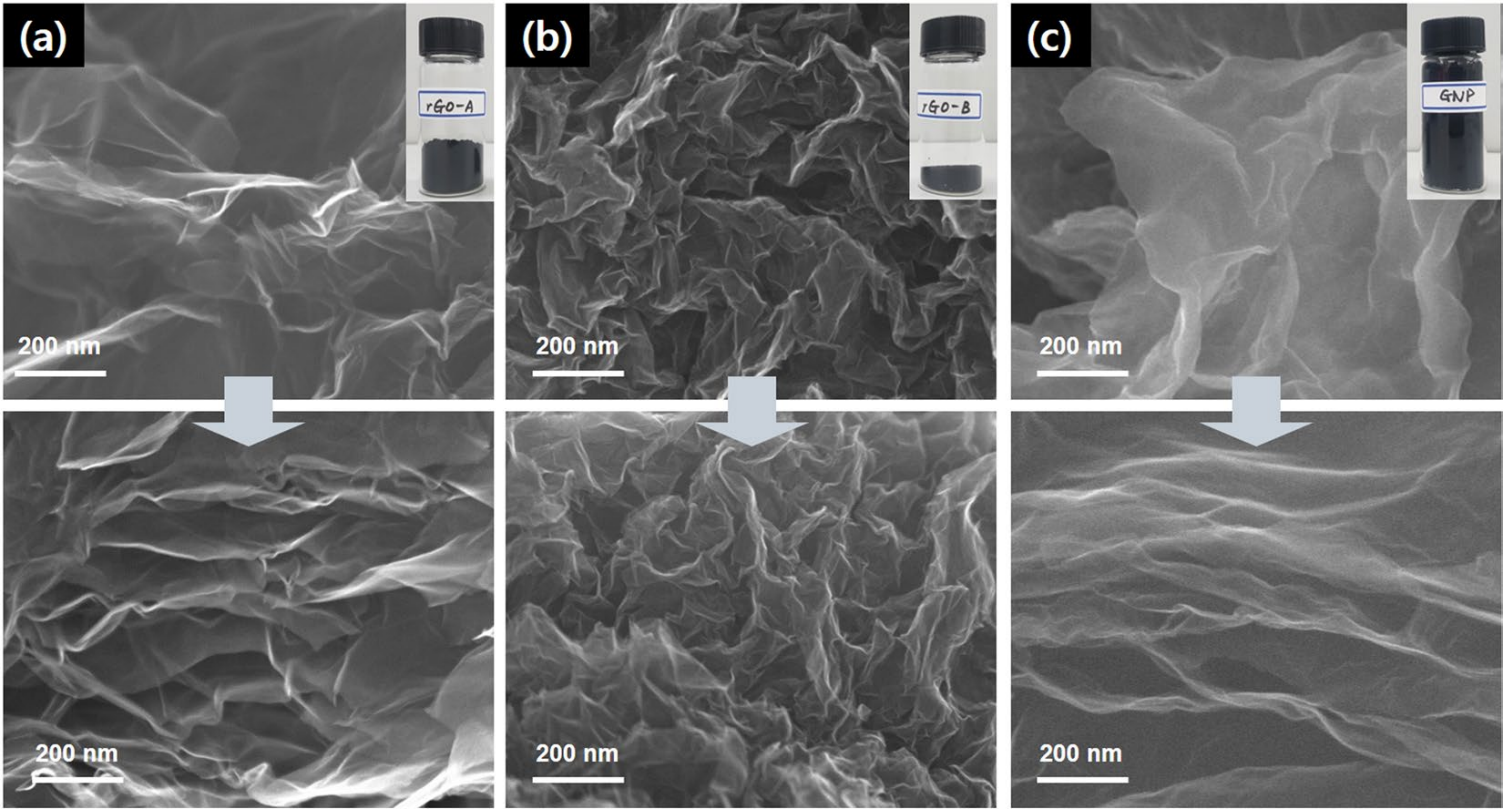
SEM images of before (top) and after (bottom) the pressurization of (**a**) rGO-A, (**b**) rGO-B, and (**c**) GNP. Insets show the photographs of the corresponding powder-type graphenes in 20 mL vials; rGO-A (0.2 g), rGO-B (1.0 g), GNP (0.1 g).

The morphology of the three samples shown in Fig. 2 was observed by scanning electron microscopy (SEM). The insets of Fig. 2 show the photographs of the three types of graphene flake with 0.2 g of rGO-A, 1.0 g of rGO-B, and 0.1 g of GNP in 20 mL vials, respectively. As observed from the SEM images, rGO-A has a fluffy morphology, and occupies a larger volume despite its much smaller weight compared to the fine granule-typed rGO-B. The SEM images show that rGO-B is more wrinkled and more densely packed between the layers in the graphene flakes than is rGO-A (Fig. 2a,b). The GNP sample shows more fluffy-ash-type morphology with lower density than does the rGO-A (Fig. 2a,c). The morphologies of the samples after the pressurization up to 52 MPa are shown in Fig. 2 (bottom). Usually, it is observed that the corrugation among the flakes is enhanced by pressurization and can be correlated to the conductivities of the samples. A critical deformation via the pressurization is not observed in the microscopic study in any of the samples.

**Figure 3:**
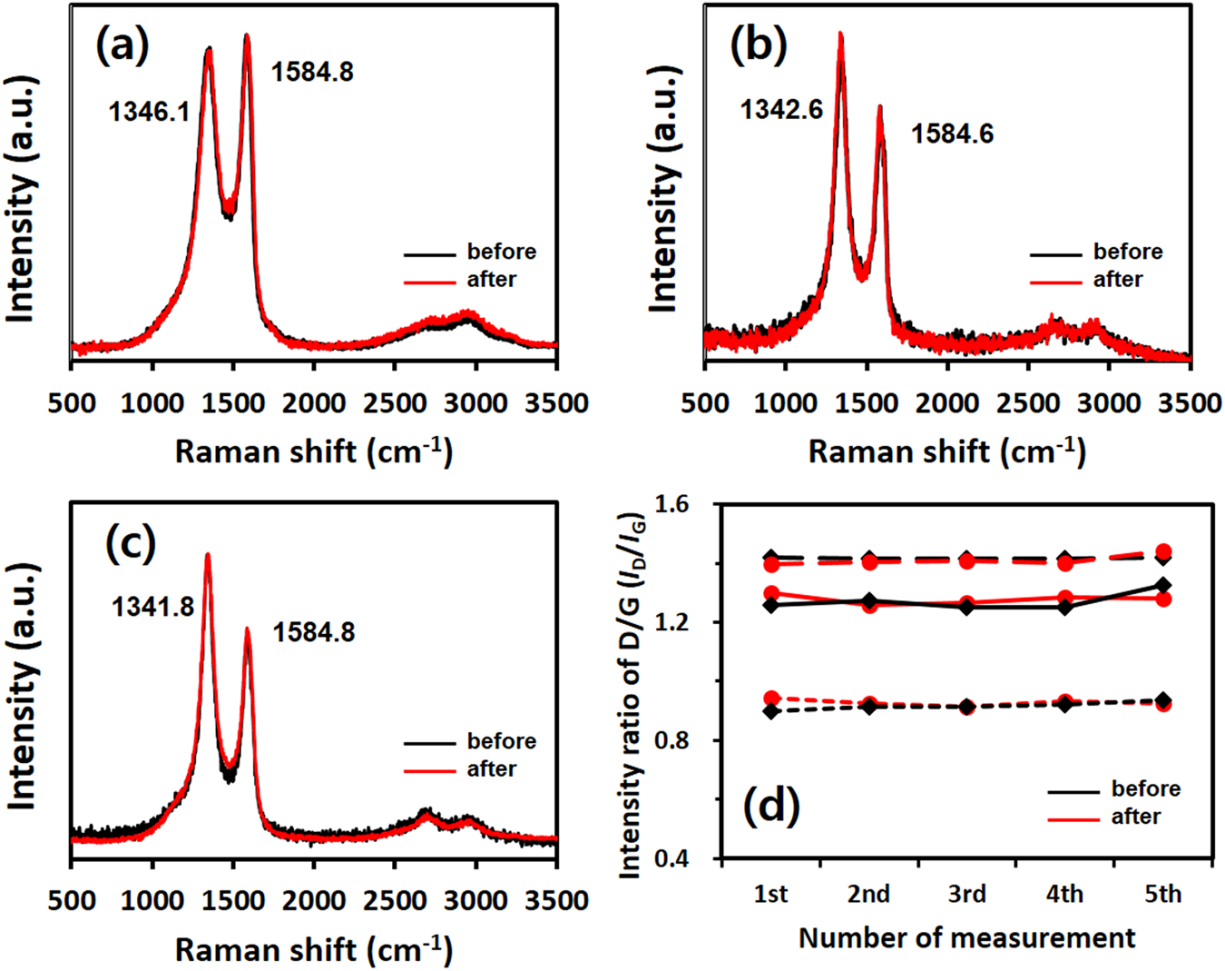
Raman spectra of (**a**) rGO-A, (**b**) rGO-B, and (**c**) GNP before (black line) and after (red line) pressurization. (**d**) Comparison data for *I*_D_/*I*_G_ of rGO-A (short-dash line), rGO-B (solid line) and GNP (long-dash line) before and after pressurization.

The structural changes in the graphene sample before and after the pressurization, which can modify the electrical conductivity of graphene are investigated by Raman spectroscopy. Figure 3a–c display the representative Raman spectra of the three graphene flakes before and after the pressurization where the intensity of each peak was normalized to the G-band intensity. The Raman measurements were carried out at five different locations for each sample. The Raman spectra of the samples exhibit tangential breathing modes (G-band) at 1585 cm^−1^ and disorder modes (D-band) at approximately 1340 cm^−1^ ^32^. Since the D/G ratio (*I*_D_/*I*_G_) is generally considered to be a measure of the defect density of carbon materials containing sp^2^ bonding^32,33^, possible structural deformations before and after pressurization were examined through the changes in the D/G ratios. A significant variation in the ratio was not observed in the three types of graphene flakes (Fig. 3d). Thus, the structural stability of the samples before and after the pressurization was confirmed.

After confirming by microscopic and spectroscopic methods that no structural changes were present in the samples, electrical measurements on the three types of graphene flake samples were conducted to obtain several parameters, such as sheet resistance, resistivity, and conductivity. The schematic illustration and images of the measurement system are depicted in Fig. S2. Table S1 displays an example of the series of the measurement parameters, such as applied pressure (*P*), pellet thickness (*t*), pellet density (*d*_*v*_), sheet resistance (*ρ*_*s*_), resistivity (*ρ*) and conductivity (*σ*) of the sample, with 0.2 g of rGO-A obtained by the measurement system. The resistance and pellet thickness values at the given applied pressure were measured using a digital multimeter and a thickness gauge, respectively. The surface resistance (*ρ*_*s*_) was calculated by multiplying the resistance (*R*) by the geometrical correction factor (*F*) $({\rho }_{s}=R\times F)$. Considering the dimensions of the probe head shown in Fig. S2, the geometrical correction factor of 4.294 was applied for the *ρ*_*s*_ calculation^29^. The resistivity (*ρ*) and conductivity (*σ*) of the pellet are calculated by Eqs (1) and (2)^29,30^ as follows:

1 $$\rho =R\times F\times t$$

2 $$\sigma =\frac{1}{\rho }$$

where *R* is the electrical resistance, *t* is the thickness of the specimen (pellet) measured by the thickness gauge in the apparatus, and *F* is a geometrical correction factor.

**Figure 4:**
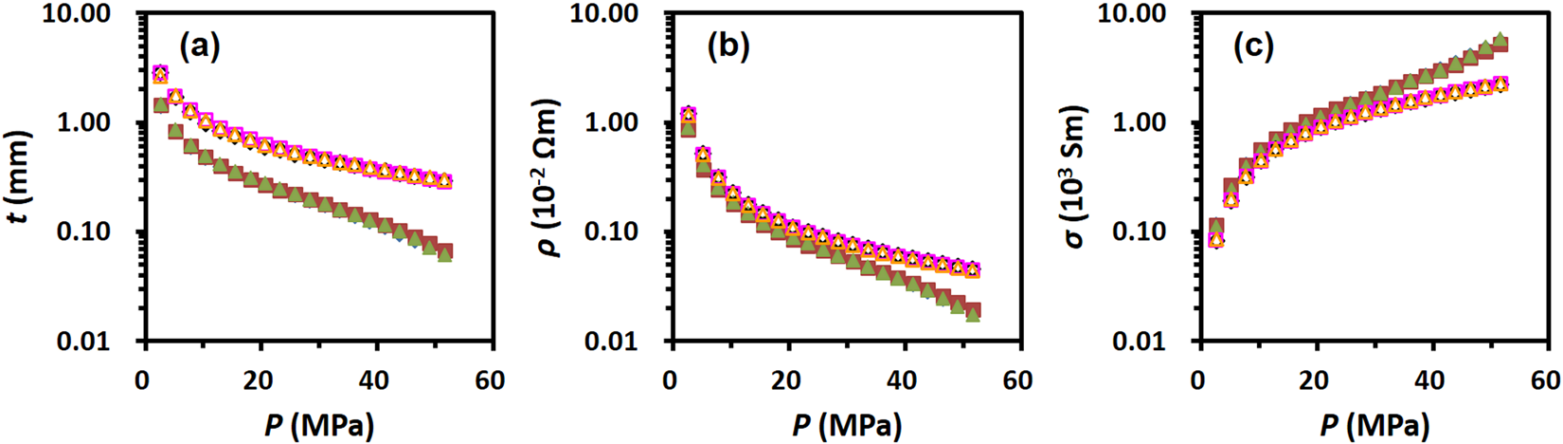
Correlation plots of (**a**) thickness (*t*), (**b**) resistivity (*ρ*), and (**c**) conductivity (*σ*) as a function of the applied pressure (*P*); 0.1 g (filled symbol) and 0.2 g (unfilled symbol) of rGO-A. Each colour represents data accomplished on different sampling.

The thickness as a function of the applied pressure was then measured in order to determine the intrinsic electrical behaviour the samples. Since the thickness of a sample pellet at the applied pressure depends on the dose of the sample, thickness measurements were performed on two different pellets made with 0.1 g and 0.2 g of rGO-A at the initial test. The plots of the thickness, resistivity, and conductivity as a function of the applied pressure are shown in Fig. 4. Each data set in Fig. 4 exhibits good reproducibility for both pellets. As the applied pressure increases, the pellet thickness decreases because the sample is densely compacted in the cylinder (Fig. 4a). The decrease in the thickness leads to a decrease in the resistivity and an increase in the conductivity (Fig. 4b,c). As the sample amount increases, the thickness of the pellet and the resistivity at the given applied pressure increase (unfilled symbols in Fig. 4a,b), while the conductivity decreases (unfilled symbols in Fig. 4c). Compared to the similar trend observed for the correlation between the pellet thickness and the applied pressure for both doses of the sample shown in Fig. 4a, the correlations between the resistivity (or conductivity) and the applied pressure show different trends at the pressures above 40 MPa (Fig. 4b,c). Hence, it is better to determine an electrical parameter other than resistivity (or conductivity) that is independent of the sample thickness.

**Figure 5:**
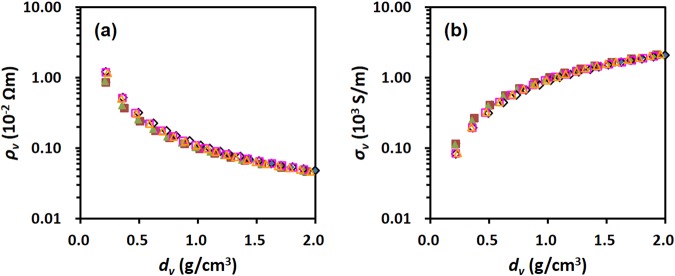
Correlation plots of (**a**) volume resistivity (*ρ*_*v*_) and (**b**) volume conductivity (*σ*_*v*_) as a function of the pellet density (*d*_*v*_); 0.1 g (filled symbol) and 0.2 g (unfilled symbol) of rGO-A. Each colour represents data obtained from a different sample.

Figure 5 shows the plot of the resistivity and conductivity of the two pellets as a function of the pellet density. The two graphs obtained for two different doses of the samples became identical, which is not observed in Fig. 4. This implies that the resistivity or conductivity obtained for each density is an intrinsic property, because the volume is the only variable parameter in the formula for the pellet density based on the following. The density of a pellet (*d*_*v*_) at a given applied pressure is calculated from the mass of the pellet and its geometric dimensions as described in Eq. (3)^25^ as follows:

3 $${d}_{v}=\frac{m}{V}=\frac{m}{A\,\times \,t}$$

where *m* is the mass of the specimen, *V* is the volume of the pellet, *A* is the cross-sectional area of the piston, and *t* is the thickness of the specimen. Since *m* and *A* in Eq. (3) were fixed in the measurement, the only variable parameter in the formula for the density was the pellet volume. Hence, it was concluded that volume resistivity (*ρ*_*v*_) or volume conductivity (*σ*_*v*_), which is considered taking the geometric dimensions into account, is an intrinsic property of a graphene flakes.

This was also found in the analysis of the graphene nanopowder (GNP) sample. Figure S3 displays the plot of the thickness, resistivity and conductivity of the two different pellets made of 0.1 g and 0.2 g of GNP as a function of the applied pressure, showing a different characteristic trend in the high-pressure area (>40 MPa). On the other hand, the comparison plots of the volume resistivity/volume conductivity as a function of the pellet density between the two pellets show identical trends, implying that their electrical properties are intrinsic properties of the material (Fig. S4).

**Figure 6:**
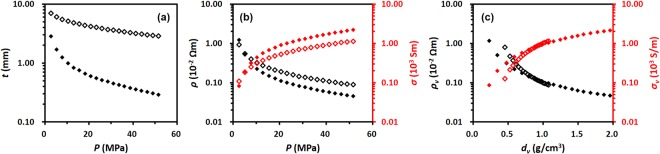
Correlation plots of (**a**) thickness (*t*) and (**b**) resistivity (*ρ*, black symbol) and conductivity (*σ*, red symbol) as a function of the applied pressure (*P*). (**c**) Volume resistivity (*ρ*_*v*_, black symbol) and volume conductivity (*σ*_*v*_, red symbol) as a function of the pellet density (*d*_*v*_). 0.2 g of rGO-A (filled symbol) and 1.0 g of rGO-B (unfilled symbol).

Electrical analyses of rGO-B (1.0 g) were performed, and the applied pressure and the pellet density dependent measurement parameters are displayed in Figs S5 and S6, respectively. Figure 6 shows the comparison of rGO-A and rGO-B. Since the mass of rGO-B is much larger than that of rGO-A (0.2 g), the rGO-B thickness values are higher under the same applied pressures (Fig. 6a), resulting in a different changes in the resistivity (and conductivity) plots for the two rGO samples (Fig. 6b). As mentioned above, the measured thickness and resistivity/conductivity parameters are not intrinsic parameters. When the volume is taken into account, the differences are eliminated so that volume resistivity/conductivity is confirmed to be intrinsic (Fig. 6c). This means that the two different samples of rGO show similar electrical characteristics even though the samples have different geometric dimensions.

**Figure 7:**
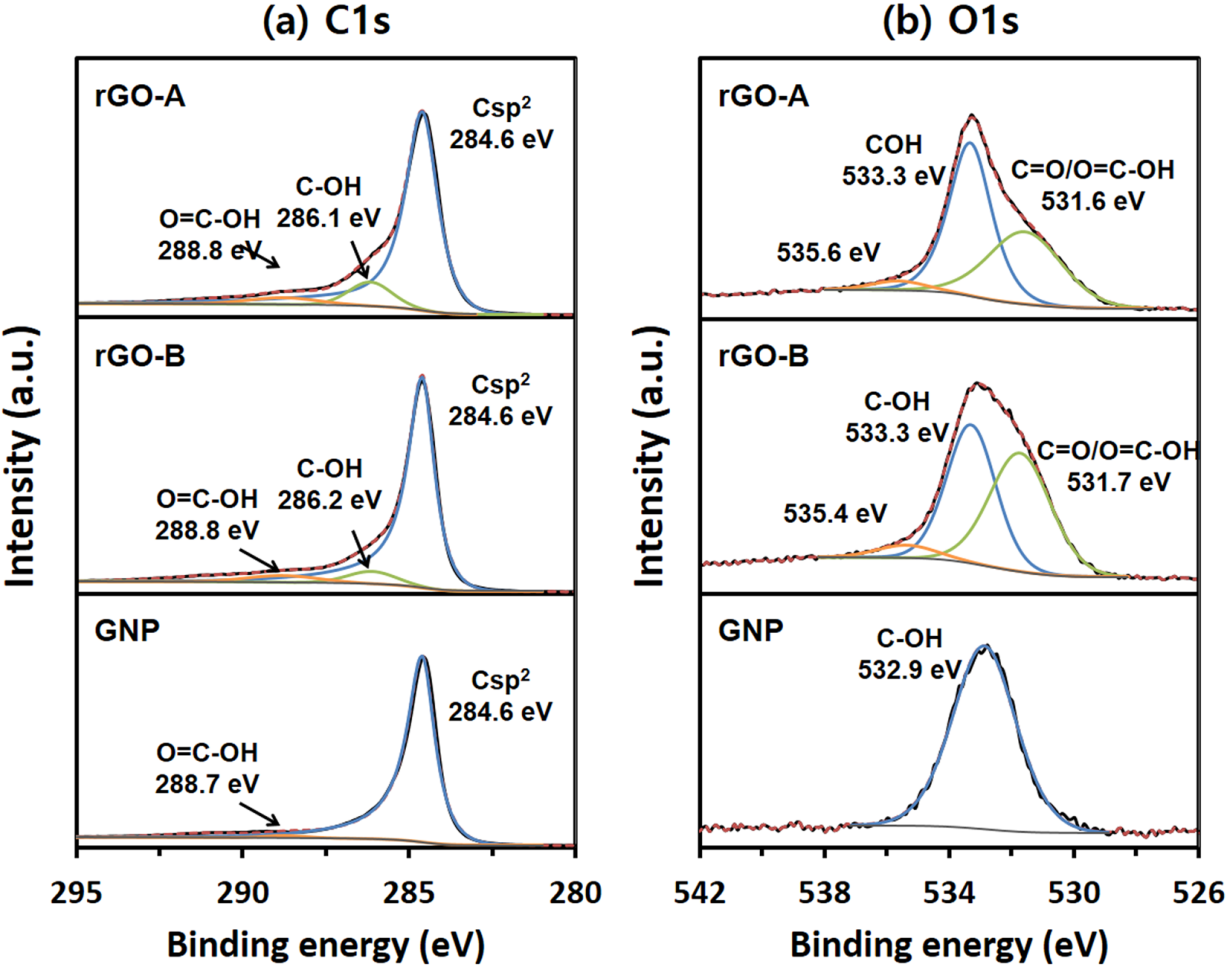
High resolution (**a**) C1s and (**b**) O1s spectra of rGO-A (top), rGO-B (middle), and GNP (bottom).

It is known that the oxygen content present in graphene materials affects their electrical conductivity with a higher oxygen content having an adverse effect on the conductivity^34,35^. To quantitatively and qualitatively analyse the chemical components in the graphene flake samples, the samples were studied by X-ray photoelectron spectroscopy (XPS). The summary of the binding energies and quantitative analysis data obtained from the survey XPS spectra are listed in Table S2. Since out of the 7 ingredient elements, carbon and oxygen were the main components in the graphene flakes, only the survey spectra of C and O are shown in Fig. S7. The atomic fractions of carbon in the rGO-A, rGO-B, and GNP, which were calculated from the measured peak areas, were 86.11%, 86.09%, and 94.28%, respectively while those of oxygen were 13.02%, 12.21%, and 4.27%, respectively. The two rGOs have similar contents of the carbon and oxygen components while the GNP sample contains a higher carbon content and a lower oxygen content than the rGOs. High-resolution XPS studies were carried out to analyse their chemical structures. The high-resolution XPS spectra of C1s of the three samples show a sharp peak at 284.6 eV corresponding to the sp^2^ carbon in the graphite component (Fig. 7a). The C1s XPS spectra of the rGO-A and rGO-B have similar peak positions and shapes. Two broad peaks were observed at 286.1 and 288.8 eV that are attributed to C-OH and O=C-OH, respectively, and are expected to be generated during chemical exfoliation^2,33,34^. The peak at 288.8 eV is also observed in the GNP spectra even though its intensity is very small, and the peak is broad. However, the peak at 286.1 eV cannot be observed in the GNP spectra. For the O1s spectra, two types of surface oxygen species can be commonly distinguished in rGO-A and rGO-B as shown in Fig. 7b. The binding energies of 533.3 and 531.6 eV are ascribed to the C-OH and C=O/O=C-OH groups, respectively^33^. On the other hand, the O1s spectrum of GNP showed only one main peak at 532.9 eV, which is assigned to C-OH. Since the carboxyl or carbonyl group is not observed in the GNP C1s spectrum, GNP contains only one oxygen type. From the above results, it is predicted that the similar content of oxygen and similar chemical state between rGO-A and rGO-B lead to the similarity of their electrical properties.

**Figure 8:**
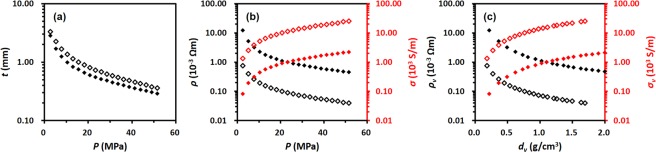
Correlation plots of (**a**) thickness (*t*) and (**b**) resistivity (*ρ*, black symbols) and conductivity (*σ*, red symbol) as a function of the applied pressure (*P*). (**c**) Volume resistivity (*ρ*_*v*_, black symbol) and volume conductivity (*σ*_*v*_, red symbol) as a function of the pellet density (*d*_*v*_). 0.2 g of rGO-A (filled symbols) and 0.2 g of GNP (unfilled symbols).

Figure 8 shows the comparison of the parameters measured for rGO-A (0.2 g) and GNP pellets (0.2 g) as the function of applied pressure and the volume density. In Fig. 8a, the GNP pellet thicknesses show higher values for a given applied pressure because the GNP has a lower density than rGO-A, as shown in the photographs of the samples (insets of Fig. 2a,c). Despite its higher film thickness, the conductivity of the GNP pellets under any applied pressure is approximately 10 times larger than that of rGO-A. The same trend in the conductivities can still be observed in the plot of the correlation between the density of the pellet and its volume conductivity. In Fig. 8c, the volume conductivity of the GNP pellet under any pellet density is much higher than that of the rGO-A pellet. Taking all results into consideration, it could be concluded that the intrinsic electrical property of the graphene flakes used in this experiment is influenced mainly by the oxygen content of the graphene structure.

## Conclusions

We elucidated the intrinsic property that can represent the electrical characteristics of graphene flakes. Plots of the resistivity or conductivity versus the applied pressure showed different curves for the different amounts of the rGO sample. This implies that these two parameters are not intrinsic for graphene flakes. Meanwhile, the plots of the volume resistivity or volume conductivity versus the pellet density showed identical curve that were independent of the sample amount. Similar results were also obtained for the GNP samples. Hence, it is the volume resistivity or volume conductivity that can represent the intrinsic electrical property of a graphene flake. In addition, XPS studies confirmed that the intrinsic electrical property of the graphene flake is mainly influenced by the contents of carbon and oxygen in the graphene structure. It is believed that our results will be useful for evaluating the electrical quality of the graphene flakes and can be further applied to other 2D conducting materials.

## Methods

### Materials

As a powder-type graphene product, two kinds of reduced graphene oxide (rGO-A and rGO-B) were purchased from Graphene Supermarket Inc., (USA) and Graphenea Inc. (Spain), respectively. Graphene nanopowder (GNP, average flake thickness: 1.6 nm) was also obtained from Graphene Supermarket Inc. (USA). The powder-type graphene materials were used after vacuum drying at 80 °C for 24 hours in order to remove the remaining water content in the sample.

### Measurement apparatus

A powder resistivity measurement system (Hantech Co., Ltd. Korea) was used for the volume resistivity measurements. The measurement system consists of two components: i) a pelletizer and electrode unit and ii) an electrical measurement system. The details of the measurement system are shown in Fig. S1. The pelletizer is composed of a 4-probe head, a piston, and a cylinder. The head of the piston and the inside of the cylinder are covered with a non-conducting zirconia material, so that the specimen is electrically isolated from all sides. The 4-probe setup consists of four equally spaced gold rods with identical radius and each gold rod is embedded in a zirconia template. The inner diameter of the cylinder is 20.4 mm, the probe spacing is 1.6 mm and the diameter of the four electrodes is 1.4 mm. The applied pressure is measured by a pressure gauge with high precision for a maximum pressure of 52 MPa.

### Measurement procedure

A dose of the pretreated graphene flake in a vacuum oven, ranging from 0.1 g to 1 g, was transferred into a pelletizer. First, the 4-probe head (ii) and the cylinder (iii) were assembled by inserting the former into the latter and the pretreated sample was charged inside the cylinder. Then, after tapping the sample cylinder in order to obtain a uniform distribution of the powder for a flat surface, the piston (i) was inserted in the cylinder charged with the sample. Finally, the sample-charged pelletizer was connected to the electrode unit, and then pressure varying from 2.6 to 52 MPa was applied to the pelletizer. While pressurizing, the resistance values were recorded at the given pressures using the 4-probe system. Simultaneously, the pellet thickness was measured for the calculation of the pellet’s resistivity. The measurements were performed on three different samples to confirm the reliability and reproducibility of the data sets and between the data sets.

### Characterization

The morphologies of graphene flake before and after applying the pressure were characterized by ultrahigh-resolution field emission scanning electron microscopy (UHR FE-SEM) using a Hitachi S-5500 instrument and by field emission-transmission electron microscopy (FE-TEM) using a JEOL JEM 2100 F instrument. The structural characteristics were investigated using Raman spectroscopy (NT-MDT, Ntegra Spectra Duo Max) that was performed at an excitation wavelength of 532 nm. X-ray photoelectron spectroscopy (XPS) studies were performed using a K-ALPHA+ (Thermo Scientific, UK) system with an aluminium anode (Al K*α*, 1486.6 eV) at 12 kV and 72 W.

## Supplementary information

Supplementary Information

## Data Availability

All data included in this study are available upon request by contact with the corresponding author.
