# Supplementary material for: Elucidation of an intrinsic parameter for evaluating the electrical quality of graphene flakes
Source: Sci Rep. 2019 Jan 24;9:557. doi: 10.1038/s41598-018-37010-x (PMC6346114; doi:10.1038/s41598-018-37010-x)
Supplement: Supplementary file 1 — Supplementary Information [file 41598_2018_37010_MOESM1_ESM.pdf]

Elucidation of an intrinsic parameter for evaluating the electrical quality of graphene flakes

Ha-Jin Lee[1,2],* Ji Sun Kim[1,2], Kwang Young Lee[3], Kyung Ho Park[4], Jong-Seong Bae[5], and Mahfuza Mubarak[1,2] & Haeseong Lee[3]*

[1]*Western Seoul Center, Korea Basic Science Institute, 150 Bugahyun-ro, Seoudaemun-gu, Seoul 03759, Republic of Korea, E-mail: hajinlee@kbsi.re.kr*

[2]*Department of Chemistry and Nano Science, Ewha Womans University, 52 Ewhayeodae-gil, Seoudaemun-gu, Seoul 03760, Republic of Korea*

[3]*Department of Nano and Advanced Materials Engineering, Jeonju University, 303 Cheonjam-ro, Wansan-gu, Jeonju 55069, Republic of Korea, E-mail: haeseong@jj.ac.kr*

[4]*Korea Advanced Nano Fab Center, 109 Gwanggyo-ro, Yeongtong-gu, Suwon 16229, Republic of Korea*

[5]*Busan Center, Korea Basic Science Institute, 30 Gwahaksandan 1-ro 60beon-gil, Gangseo-gu, Busan 46742, Republic of Korea*

Table S1. An example of the measurement parameters obtained for rGO-A (0.2 g): applied pressure (*P*), pellet thickness (*t*), pellet density (d_v), sheet resistance (ρ_s), resistivity (ρ) and conductivity (σ).

p (MPa)	t (mm)	d_v (g/cm^3)	R (Ω)	ρ_s (Ω/sq)	ρ (Ωm)	σ (S/m)
2.61	2.643	0.232	1.017	4.37	1.15E-02	87
5.16	1.758	0.348	0.660	2.83	4.98E-03	201
7.74	1.279	0.478	0.558	2.40	3.07E-03	326
10.32	1.038	0.590	0.490	2.10	2.18E-03	458
12.90	0.874	0.700	0.459	1.97	1.72E-03	581
15.48	0.770	0.795	0.433	1.86	1.43E-03	699
18.06	0.687	0.891	0.419	1.80	1.24E-03	809
20.63	0.619	0.989	0.404	1.73	1.07E-03	932
23.21	0.566	1.081	0.395	1.70	9.60E-04	1,042
25.79	0.531	1.152	0.384	1.65	8.75E-04	1,142
28.37	0.491	1.246	0.377	1.62	7.95E-04	1,257
30.97	0.464	1.319	0.369	1.58	7.34E-04	1,362
33.54	0.432	1.416	0.364	1.56	6.75E-04	1,481
36.12	0.409	1.496	0.358	1.54	6.28E-04	1,593
38.70	0.387	1.581	0.353	1.52	5.87E-04	1,705
41.28	0.364	1.681	0.351	1.51	5.48E-04	1,824
43.85	0.344	1.779	0.349	1.50	5.15E-04	1,942
46.44	0.327	1.871	0.346	1.49	4.86E-04	2,056
49.02	0.312	1.961	0.344	1.48	4.61E-04	2,169
51.57	0.297	2.060	0.340	1.46	4.33E-04	2,308

Table S2. Atomic fractions of the elements calculated from the XPS peak areas.

Elements	B.E. (eV)	Atomic %		
		rGO-A	rGO-B	GNP
C 1s	284.60	86.11	86.09	94.28
Cl 2p	201.88	0.03	0.11	
N 1s	299.57	0.16	0.52	0.26
Na 1s	1071.50	0.17	0.14	0.04
O 1s	533.28	13.02	12.21	4.27
S 2p	168.41	0.21	0.30	0.81
Si2p	102.96	0.30	0.63	0.34

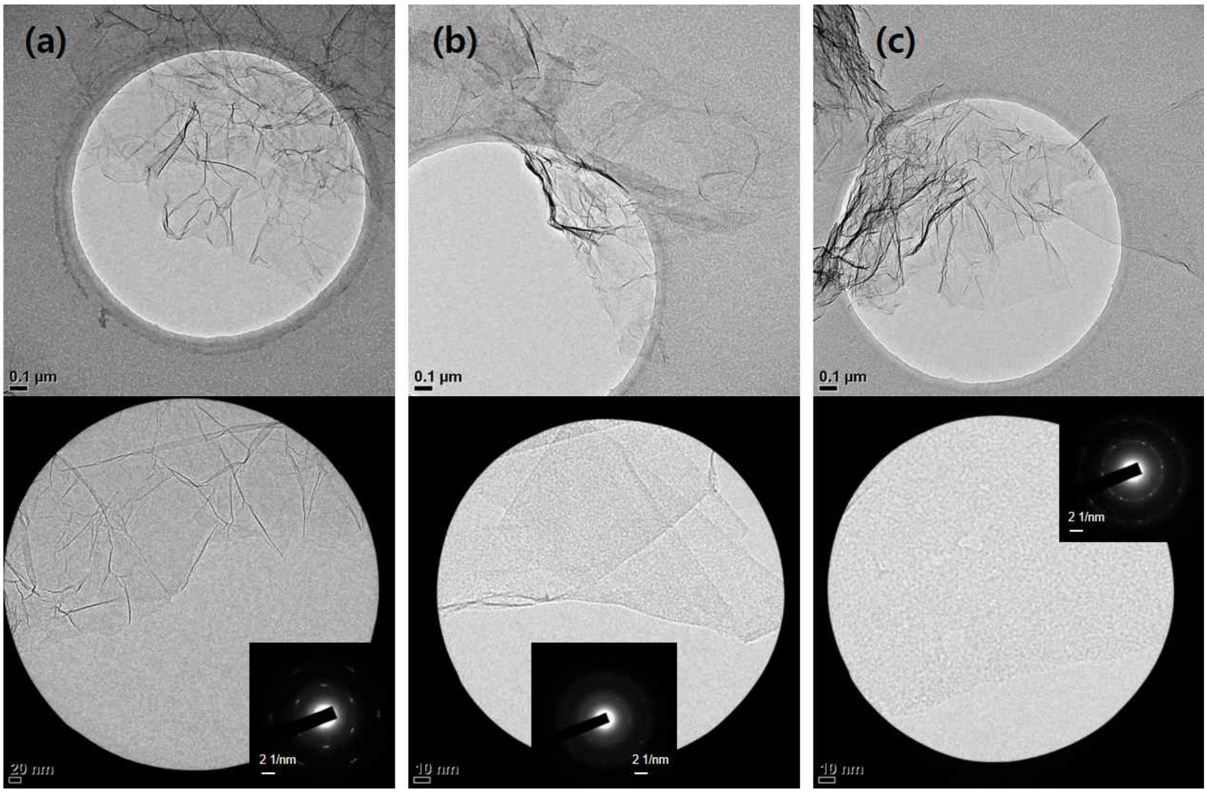

Figure S1. TEM images (top) and SAED patterns (bottom) of (a) rGO-A, (b) rGO-B, and (c) GNP.

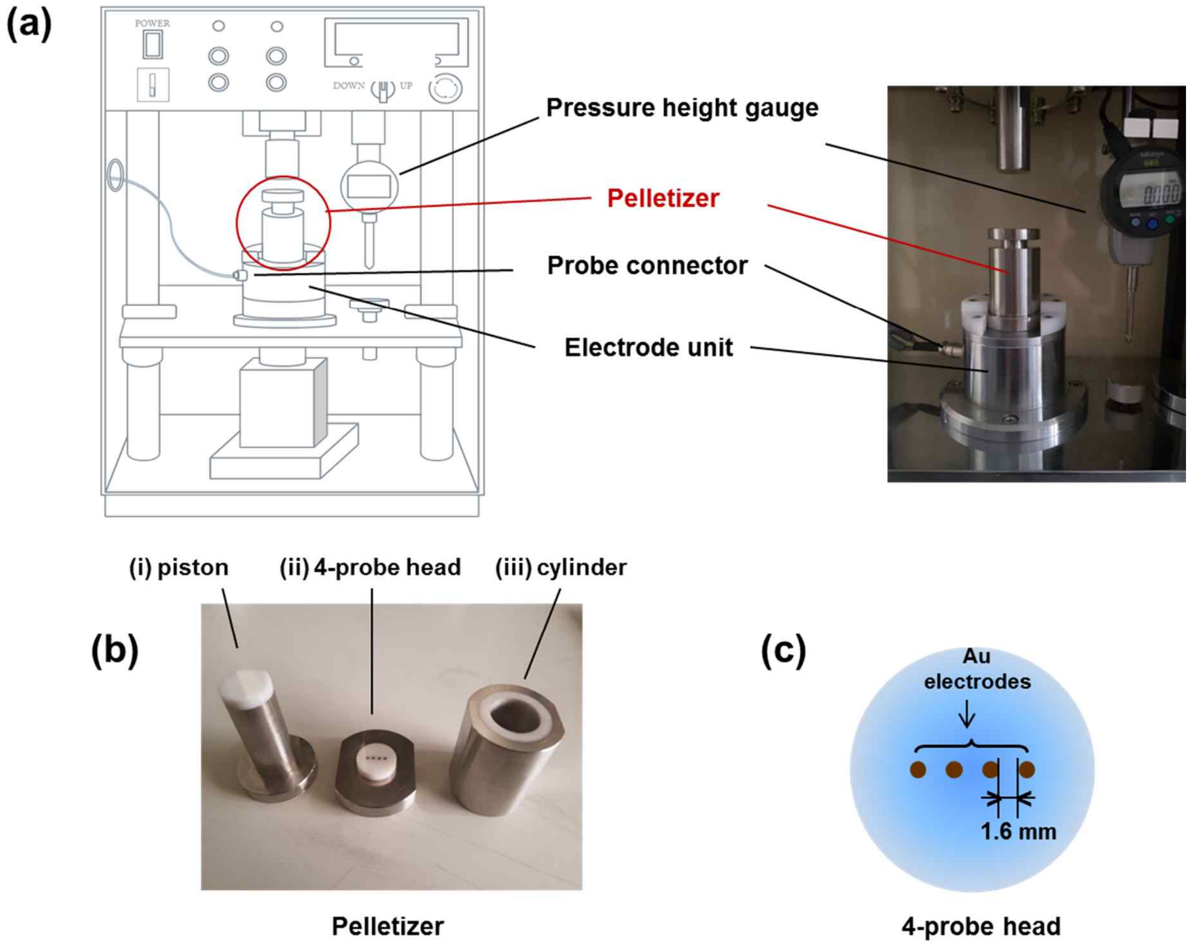

Figure S2. (a) Schematic diagram and the photograph of the powder resistivity measurement system. (b) Pelletizer equipped with a 4-probe head and piston and (c) a top view of a 4-probe head with dimension.

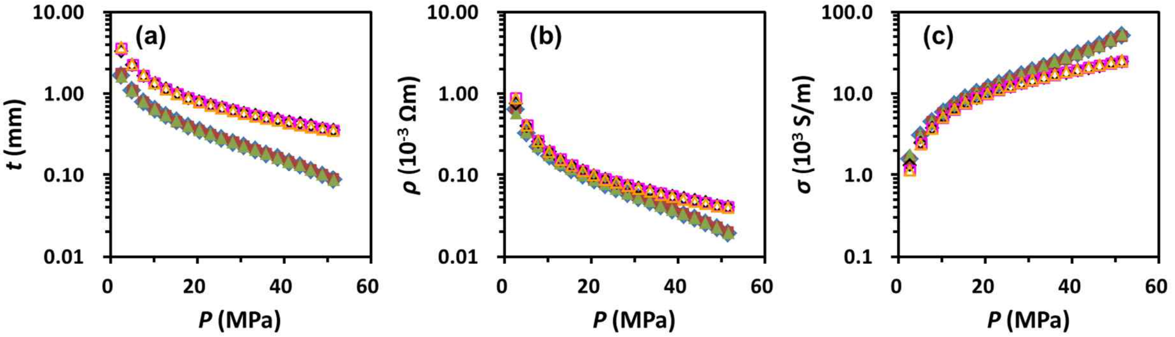

Figure S3. Correlation plots of (a) thickness (t), (b) resistivity (ρ), and (c) conductivity (σ) as a function of the applied pressure (P); 0.1 g (unfilled symbol) and 0.2 g (filled symbol) of GNP. Each colour represents data obtained from different samples.

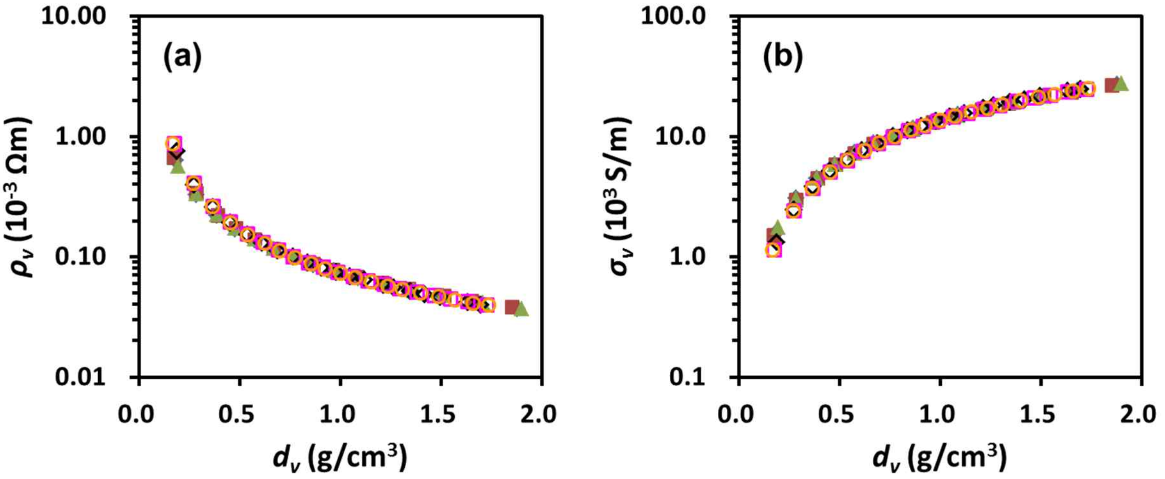

Figure S4. Correlation plots of (a) volume resistivity (ρ_v) and (b) volume conductivity (σ_v) as a function of the pellet density (d_v); 0.1 g (filled symbol) and 0.2 g (unfilled symbol) of GNP. Each colour represents data obtained from different samples.

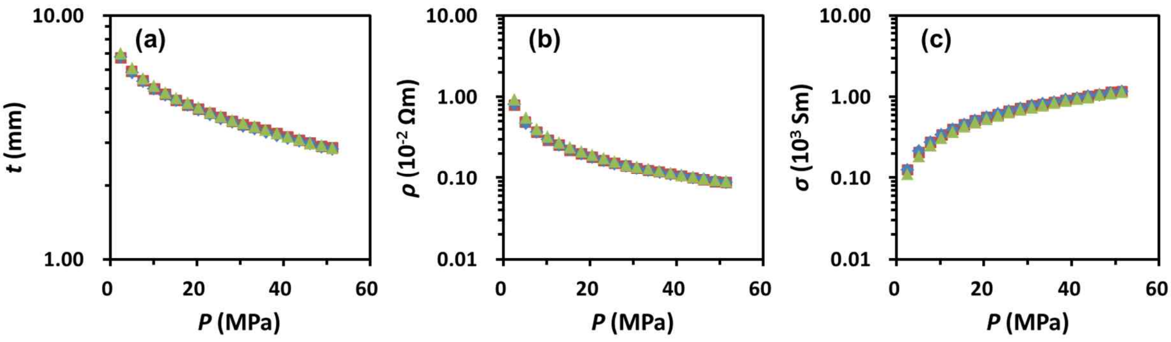

Figure S5. Correlation plots of (a) thickness (t), (b) resistivity (ρ), and (c) conductivity (σ) of rGO-B (1.0 g) as a function of the applied pressure (P). Each colour represents data obtained from different samples.

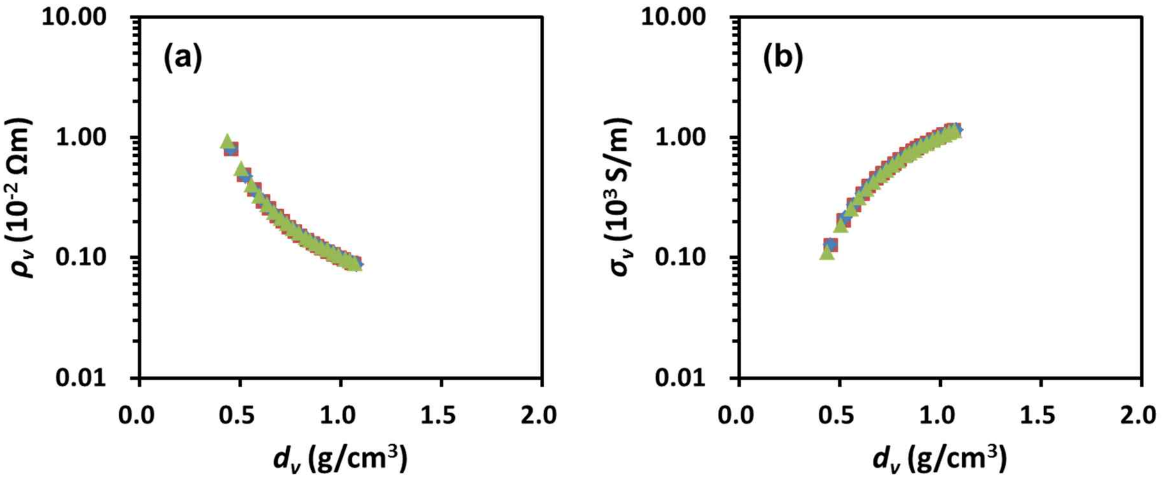

Figure S6. Correlation plots of (a) volume resistivity (ρ_v) and (b) volume conductivity (σ_v) of rGO-B (1.0 g) as a function of the pellet density (d_v). Each colour represents data obtained from different samples.

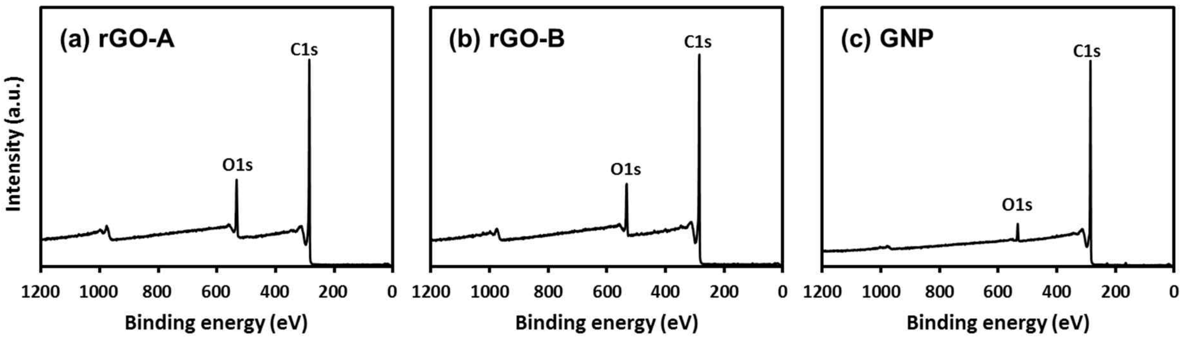

Figure S7. XPS survey spectra of (a) rGO-A, (b) rGO-B and (c) GNP.
